# Combining free and aggregated cellulolytic systems in the cellulosome-producing bacterium *Ruminiclostridium cellulolyticum*

**DOI:** 10.1186/s13068-015-0301-4

**Published:** 2015-08-13

**Authors:** Julie Ravachol, Romain Borne, Isabelle Meynial-Salles, Philippe Soucaille, Sandrine Pagès, Chantal Tardif, Henri-Pierre Fierobe

**Affiliations:** Aix-Marseille Université-CNRS, LCB UMR7283, IMM, 31 chemin Joseph Aiguier, 13402 Marseille, France; Université de Toulouse, INSA, UPS, INP, LISBP, 135 Avenue de Rangueil, 31077 Toulouse, France; INRA, UMR792 Ingénierie des Systèmes Biologiques et des Procédés, 31400 Toulouse, France; CNRS, UMR5504, 31400 Toulouse, France

**Keywords:** *Ruminiclostridium cellulolyticum*, *Lachnoclostridium phytofermentans*, Cphy_3367, Cellulosome, Free cellulase, Dockerin

## Abstract

**Background:**

*Ruminiclostridium cellulolyticum* and *Lachnoclostridium phytofermentans* (formerly known as *Clostridium cellulolyticum* and *Clostridium phytofermentans*, respectively) are anaerobic bacteria that developed different strategies to depolymerize the cellulose and the related plant cell wall polysaccharides. Thus, *R. cellulolyticum* produces large extracellular multi-enzyme complexes termed cellulosomes, while *L. phytofermentans* secretes in the environment some cellulose-degrading enzymes as free enzymes. In the present study, the major cellulase from *L. phytofermentans* was introduced as a free enzyme or as a cellulosomal component in *R. cellulolyticum* to improve its cellulolytic capacities.

**Results:**

The gene at locus Cphy_3367 encoding the major cellulase Cel9A from *L. phytofermentans* and an engineered gene coding for a modified enzyme harboring a *R. cellulolyticum* C-terminal dockerin were cloned in an expression vector. After electrotransformation of *R. cellulolyticum*, both forms of Cel9A were found to be secreted by the corresponding recombinant strains. On minimal medium containing microcrystalline cellulose as the sole source of carbon, the strain secreting the free Cel9A started to grow sooner and consumed cellulose faster than the strain producing the cellulosomal form of Cel9A, or the control strain carrying an empty expression vector. All strains reached the same final cell density but the strain producing the cellulosomal form of Cel9A was unable to completely consume the available cellulose even after an extended cultivation time, conversely to the two other strains. Analyses of their cellulosomes showed that the engineered form of Cel9A bearing a dockerin was successfully incorporated in the complexes, but its integration induced an important release of regular cellulosomal components such as the major cellulase Cel48F, which severely impaired the activity of the complexes on cellulose. In contrast, the cellulosomes synthesized by the control and the free Cel9A-secreting strains displayed similar composition and activity. Finally, the most cellulolytic strain secreting free Cel9A, was also characterized by an early production of lactate, acetate and ethanol as compared to the control strain.

**Conclusions:**

Our study shows that the cellulolytic capacity of *R. cellulolyticum* can be augmented by supplementing the cellulosomes with a free cellulase originating from *L. phytofermentans*, whereas integration of the heterologous enzyme in the cellulosomes is rather unfavorable.

**Electronic supplementary material:**

The online version of this article (doi:10.1186/s13068-015-0301-4) contains supplementary material, which is available to authorized users.

## Background

Consolidated BioProcessing (CBP) is an attractive strategy for low cost production of biofuel. Anaerobic cellulolytic bacteria are promising CBP candidates for direct conversion of lignocellulosic biomass into primary alcohol or other industrially relevant compounds, as they efficiently degrade cellulose and related plant cell wall polysaccharides while often producing some valuable chemicals [1–5]. In this respect*, Ruminiclostridium cellulolyticum* (previously known as *Clostridium cellulolyticum* [6]) displays several advantageous characteristics: this mesophilic bacterium metabolizes cellulose but also some hemicellulosic polysaccharides like arabinoxylan [7–9], and mainly produces ethanol, acetate and lactate [2, 10].

Like most anaerobic and cellulolytic bacteria, *R. cellulolyticum* synthesizes large extracellular multi-enzyme complexes called cellulosomes, which depolymerize plant cell wall polysaccharides into fermentable sugars [11, 12]. The cellulosomes produced by *R. cellulolyticum* are composed of a single primary scaffoldin which contains a powerful cellulose binding module, two X2 modules of unknown function(s) and eight copies of a receptor domain, called the cohesin which strongly interacts with a complementary module borne by the catalytic subunits and termed the dockerin [13, 14]. *R. cellulolyticum* displays a single type of functional cohesin/dockerin (Type I) docking system, which is not specific, i.e. any dockerin can bind to any of the eight cohesins of the scaffoldin with comparable affinity, though enzyme discrimination was found to occur during the cellulosome assembly [14, 15]. The cellulosomes produced by *R. cellulolyticum* may be considered as “simple” compared to other cellulolytic anaerobic bacteria like *Ruminiclostridium thermocellum* [16–18] (formerly known as *Clostridium thermocellum* [6]) or *Ruminococcus flavefaciens* which produce several interacting scaffoldins and up to five different and specific cohesin/dockerin docking systems [19–21]. Nevertheless, the genome of *R. cellulolyticum* putatively encodes 62 different dockerin-containing proteins [22], including 19 predicted cellulases whose cellulose-hydrolyzing activity was experimentally demonstrated for 14 of them [23–30]. The scaffoldin CipC and the cellulases Cel48F and Cel9E are the most abundant components [12, 24, 26, 31], but proteomic analyses have showed that up to 50 different dockerin-containing proteins can concurrently participate to the cellulolytic complexes [8, 22]. Thus, *R. cellulolyticum* secretes heterogeneous populations of cellulosomes in terms of enzymatic composition.

In contrast, the anaerobic bacterium *Lachnoclostridium phytofermentans* (formerly known as *Clostridium phytofermentans* [6]) has selected a different cellulolytic system to degrade cellulose, since this mesophilic micro-organism does not synthesize any cellulosome and secrete cellulases and related plant cell wall degrading enzymes in the free state [32, 33]. The repertoire of cellulase-encoding genes in *L. phytofermentans* is noticeably less extensive than that of cellulosome-producing bacteria. For instance, 13 different Family-9 Glycoside Hydrolase (GH9) are produced by *R. cellulolyticum* most of them being cellulosomal cellulases [25, 30], whereas *L. phytofermentans* synthesizes a single free GH9 encoded by the gene at locus Cphy_3367 [34]. Nevertheless, the latter enzyme appears essential since the inactivation of its gene abolished the ability of the recombinant strain to degrade and grow on cellulose [32, 33]. The pivotal role of this GH9 enzyme hereafter called Cel9A, was further supported by the elevated activity displayed by the purified Cel9A on different cellulose substrates [35].

In most former studies which attempted to improve the properties of *R. cellulolyticum* as a putative CBP candidate, metabolic engineering was performed to accelerate the carbon flow and/or increase the yield of valuable chemicals such as ethanol [36, 37]. In the present report, the crucial role of the free Cel9A in the cellulolytic system of *L. phytofermentans* and its elevated activity on different cellulose substrates prompted us to explore an alternative strategy to improve the cellulolytic capacity of *R. cellulolyticum*. Thus, in the present study, Cel9A was introduced in the cellulosome-producing bacterium as a free or as a cellulosomal enzyme and the ability of the recombinant strains to degrade and grow on two different crystalline cellulose substrates was explored. Finally, the impact on the metabolism in terms of alcohol and acids productions of the most cellulolytic engineered strain was also investigated.

## Results

### Characterization of wild-type and engineered forms of Cel9A from *L. phytofermentans*

**Fig. 1:**
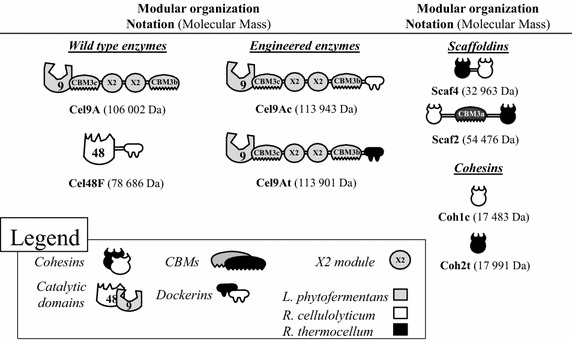
Schematic representation of the recombinant proteins used in this study. The GH- and CBM-families are indicated. Cel9Ac designates Cel9A from *L. phytofermentans* bearing a *R. cellulolyticum* dockerin. Cel9At designates Cel9A from *L. phytofermentans* appended with a *R. thermocellum* dockerin.

The free cellulase encoded by the gene at locus Cphy_3367 is composed of a signal sequence, a GH9 catalytic module followed by a CBM3c, two X2 modules and a C-terminal CBM3b (Fig. 1). Indeed, as Cel9A is secreted as a free enzyme by *L. phytofermentans*, the enzyme does not harbor any dockerin [32]. The gene encoding the mature form of the enzyme was cloned in frame with six His codons at the 3′ extremity in the *E. coli* expression vector pET28a. Similarly, the genes encoding two engineered forms of Cel9A bearing either a C-terminal *R. cellulolyticum* or *R. thermocellum* dockerin (Fig. 1) to produce the recombinant enzymes Cel9Ac and Cel9At, respectively, were cloned in the same vector. The three recombinant forms of Cel9A were purified and assayed on soluble (CarboxyMethyl Cellulose, CMC) and microcrystalline (Avicel) cellulose. As shown in Table 1, grafting a C-terminal dockerin had no impact on the CMCase activity of Cel9A, whereas the Avicelase activities of the engineered forms were reduced by 25–30% with respect to the wild-type cellulase. The wild-type Cel9A displayed a medium CMCase activity compared to formerly characterized cellulases from *R. cellulolyticum*, such as Cel9U, which is nearly sixfold more active than the *L. phytofermentans* cellulase on this substrate [25]. Nevertheless, wild-type Cel9A was found 3.2-fold more active on Avicel than the best cellulase characterized to date (Cel9E) originating from *R. cellulolyticum* in the same experimental conditions [25, 26].

**Table 1:** Activity of wild-type and engineered forms of Cel9A on soluble and microcrystalline cellulose

Enzyme	CMC	Avicel
Cel9A	1214 ± 44^a,b^	236 ± 1.6^b,c^
Cel9Ac	1284 ± 12	177 ± 1.4
Cel9At	1387 ± 54	164 ± 0.4

Assays were performed at 37°C with 1% (w/v) and 0.35% (w/v) of substrate for CMC and Avicel, respectively.

^a^Values are µmol of product released per µmol of enzyme min^−1^.

^b^Average and standard deviation of two independent experiments.

^c^Values are µmol of released products after 24 h of incubation with 0.1 µmol of enzyme.

**Fig. 2:**
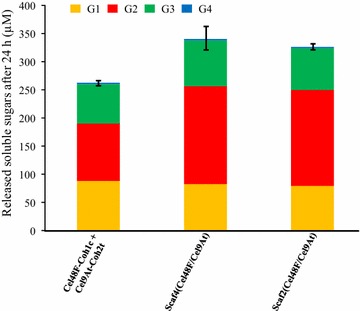
Activity on microcrystalline cellulose of bi-functional hybrid cellulosomes containing Cel9At and Cel48F. The amount of released soluble cellodextrins and their proportion by 0.1 µM of complexes or Cel9At and Cel48F bound to the corresponding free cohesins after 24 h of incubation at 37°C with 3.5 g/L Avicel are shown. Soluble sugars were identified and quantified by HPAEC-PAD. G1, G2, G3 and G4 designate glucose, cellobiose, cellotriose and cellotetraose, respectively. The data show the mean of three independent experiments and *bars* indicate the standard deviation.

The ability of Cel9A to function as a cellulosomal component was also investigated by combining Cel9At (the engineered form bearing a *R. thermocellum* dockerin) with the prominent and critical cellulosomal *R. cellulolyticum* cellulase Cel48F in bi-functional minicellulosomes [38, 39]. Cel9At and Cel48F bound onto free cohesins Coh2t and Coh1c (Fig. 1), respectively, did not display any synergy nor competition on crystalline cellulose Avicel. As shown in Fig. 2, when the enzyme pair was bound onto the hybrid scaffoldin Scaf4 (Fig. 1) which lacks a CBM3a, the resulting complex was approx. 30% more active than the corresponding free cohesin system, thereby indicating that complexation induced a moderate but significant synergy between the two cellulases, triggered by their physical proximity within the minicellulosome. However, conversely to most *R. cellulolyticum* enzyme pairs formerly tested [38], the binding of Cel9At and Cel48F onto Scaf2 which hosts a powerful CBM3a (Fig. 1), failed to enhance the overall activity compared to the Scaf4-based complex (Fig. 2), thus indicating that the “substrate targeting effect” due to the CBM3a of the scaffoldin did not promote the efficiency of this particular enzyme pair. In all configurations, the most abundant cellodextrin released was cellobiose (39–51%) followed by glucose (24–33%) and cellotriose (22–26%). Trace amounts of cellotetraose (<1%) were also detected, especially at the beginning of the kinetics.

The elevated activity of both the wild type and the engineered forms of Cel9A on crystalline cellulose prompted us to introduce the *L. phytofermentans* cellulase in *R. cellulolyticum* to improve the cellulolytic capacities of the cellulosome-producing bacterium.

### Heterologous expression of *cel9A* and *cel9Ac* in *R. cellulolyticum*

The gene encoding the wild-type Cel9A (*cel9A*) and the gene coding for the engineered form of Cel9A appended with a C-terminal *R. cellulolyticum* dockerin (*cel9Ac*) were cloned in the expression vector pSOS952, downstream of the constitutive promoter P_*thl*_ [9]. In both constructs, six His codons were introduced at the 3′ extremity of the gene. The corresponding vectors pCel9A and pCel9Ac, as well as the empty expression vector p0, were transferred into *R. cellulolyticum* using electrotransformation.

**Fig. 3:**
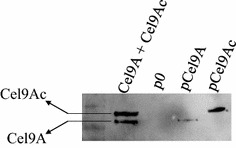
Secretion of the various forms of Cel9A by the recombinant *R. cellulolyticum* strains. The various forms of Cel9A were detected by western blot using an antiserum raised against the C-terminal 6 His tag of the heterologous enzymes, on the supernatant of cellobiose-grown cultures. The lane labeled p0 designates the supernatant of the control strain carrying the empty expression vector p0. The lanes labeled pCel9A and pCel9Ac designate the supernatants of the recombinant strains carrying pCel9A and pCel9Ac, respectively. The lane labeled “Cel9A + Cel9Ac” corresponds to a mix of Cel9A and Cel9Ac purified from *E. coli* overproducing strains.

The three isolated recombinant strains were grown on cellobiose-containing mineral medium, and exhibited similar growth and doubling times (10.8 ± 0.7 h). The capacity of *R. cellulolyticum* to secrete both forms of Cel9A was also investigated by western blot analysis of the supernatant of cellobiose-grown cultures. As shown in Fig. 3, wild-type Cel9A and Cel9Ac were detected in the medium at the appropriate mass compared to the control, thereby showing that the *L. phytofermentans* signal sequence was recognized by *R. cellulolyticum*.

### Degradation of filter paper by the recombinant *R. cellulolyticum* strains

**Fig. 4:**
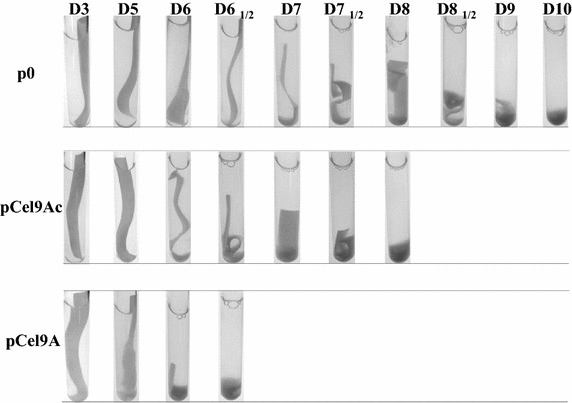
Degradation of filter paper by recombinant strains of *R. cellulolyticum* carrying p0, pCel9Ac and pCel9A. Hungate tubes containing mineral media supplemented with a stripe of filter paper at 7 g/L were inoculated (at a dilution of 1/33) with cellobiose-grown cultures of the various recombinant strains. The experiments were performed four times and snapshots were taken at least once a day. The most representative series of snapshots is shown for each strain. The duration of incubation expressed in days is indicated on top of the snapshots.

The three recombinant strains were first grown on minimal medium containing 2 g/L cellobiose until OD_450_ reached 0.8. These cultures on soluble sugar served to inoculate (1/33) a mineral medium containing filter paper at 7 g/L as the sole source of carbon and energy, and snapshots of the Hungate tubes were taken at least once a day. Four biological replicates were performed, and a representative example for each strain is shown in Fig. 4. In the case of the control strain carrying the vector p0, the aspect of the paper stripe started to change at day 7–7.5, and turned to a gel/slurry at day 10. These alterations of the paper strip seemed to occur earlier for the recombinant *R. cellulolyticum* strains producing Cel9A, especially in the case of the strain secreting the free (wild-type) form of Cel9A, since the gel aspect of the paper appeared at day 6.5, in other words approx. 3.5 days earlier compared to the control strain. For the strain secreting the cellulosomal form of Cel9A, the marked modification of the paper stripe occurred roughly 2 days in advance, with respect to the control strain.

These observations suggest an enhanced capacity of the recombinant *R. cellulolyticum* strains producing Cel9A to degrade crystalline cellulose compared to the control strain. Nevertheless, due to the nature of the selected cellulosic substrate, the improvement could not be accurately quantified.

### Growth of the various *R. cellulolyticum* strains on microcrystalline cellulose

The ability to grow on and degrade microcrystalline cellulose of the various recombinant strains was assayed similarly but using minimal medium containing 5 g/L microcrystalline cellulose Sigmacell 20 as the source of carbon and energy. Four biological replicates were performed for each strain, and samples were taken at various incubation times. As mentioned above, cultures of the three recombinant strains on 2 g/L cellobiose (OD_450_ = 0.8) served to inoculate the cellulose-containing mineral medium with exactly the same quantity of cells at the same growth phase. In addition, the inoculum was at 1/90 (v/v) to keep the residual cellobiose concentration brought by the cellobiose-grown inoculum below 10 mg/L. The bacterial growth was determined by quantification of the total proteins after centrifugation of the sample using the Lowry method [40]. The sample pellet was also used to estimate the residual cellulose content after total hydrolysis into glucose using sulfuric acid (see “Methods”) and subsequent determination of the amount of glucose by HPAEC-PAD [25]. The amount and nature of soluble sugars that might be present in the supernatant of each sample were also monitored using HPAEC-PAD.

**Fig. 5:**
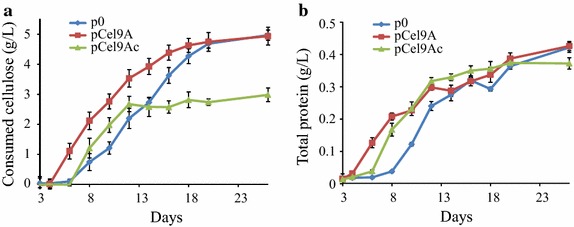
Cellulose consumption (**a**) and growth (**b**) of the various *R. cellulolyticum* strains on microcrystalline cellulose. Forty-five-mL mineral media supplemented with Sigmacell 20 microcrystalline cellulose (5 g/L) was inoculated (1/90) with cellobiose-grown cultures of the various recombinant strains. Samples were taken at specific times, centrifuged and the pellet was analyzed for residual cellulose content by HPAEC-PAD after complete hydrolysis into glucose using sulfuric acid, and total protein content using the Lowry method. *Blue line* and *diamonds* designate the control strain carrying p0; *red line* and *squares* correspond to the recombinant strain carrying pCel9A; *green line* and *triangles* designate the recombinant strain carrying pCel9Ac. The data show the mean of four independent experiments and *bars* indicate the standard deviation.

The cellulose consumption and bacterial growth for each strain are shown in Fig. 5a, b, respectively. As expected, only trace amounts of cellobiose (<30 µM) were detected in the first 2 days of culture and brought by the cellobiose-grown inoculum, but no cellodextrin or glucose were subsequently detected in the supernatants even after 26 days of incubation, in all cases. This result indicates that for all strains, the soluble sugars produced by the hydrolysis of the cellulose were rapidly consumed by the bacteria, even during the stationary phase. As reported in Fig. 5a, a significant cellulose consumption by the strain secreting the free Cel9A occurred sooner than for the two other strains. Thus 50% (2.5 g/L) of the cellulose were metabolized within 9 days by this recombinant strain, whereas the control strain required 13 days to consume the same amount of cellulosic substrate. The strain carrying pCel9A was also characterized by a significantly shorter lag phase leading to an exponential phase that started approx. 4–5 days in advance compared to the control strain, and a stationary phase occurring around day 13.

Thus the presumed enhanced cellulolytic capacity of the strain secreting free Cel9A suggested by the data obtained on filter paper, was clearly confirmed by the growth on microcrystalline cellulose and its consumption by this recombinant strain of *R. cellulolyticum*.

In contrast, the strain producing the cellulosomal form of Cel9A (Cel9Ac) displayed an unexpected profile of cellulose consumption. Though up to day 12, the cellulose consumption was slightly faster than for the control strain, the degradation stopped at day 12–13 at around 50% and remained steady until the end of the incubation at day 26. Furthermore, prolonged cultivation times (up to 45 days) did not improve the rate of cellulose consumption which remained at approx. 50%. Thus, this recombinant strain was no longer able to completely metabolize 5 g/L of crystalline substrate, in contrast to the two other strains. Nevertheless, despite different rates of cellulose consumption varying from 50% (pCel9Ac) to nearly 100% (pCel9A and p0), the three recombinant strains exhibited the same final biomass (±10%) at the end of the cultivation time (Fig. 5b), and similar doubling times ranging from 21 h for the strain carrying pCel9A to 25 h for the control strain.

### Analyses of the cellulosomes and the cellulose-bound fractions

For each recombinant strain, cultures in 0.8-L of basal rich medium supplemented with 5 g/L microcrystalline cellulose were performed. After 6 days of growth, the cultures were stopped, and the residual cellulose was harvested by filtration and extensively washed to remove the cells. The cellulosomes and other proteins bound to the cellulose were eluted using distilled water prior analysis by size exclusion chromatography.

**Fig. 6:**
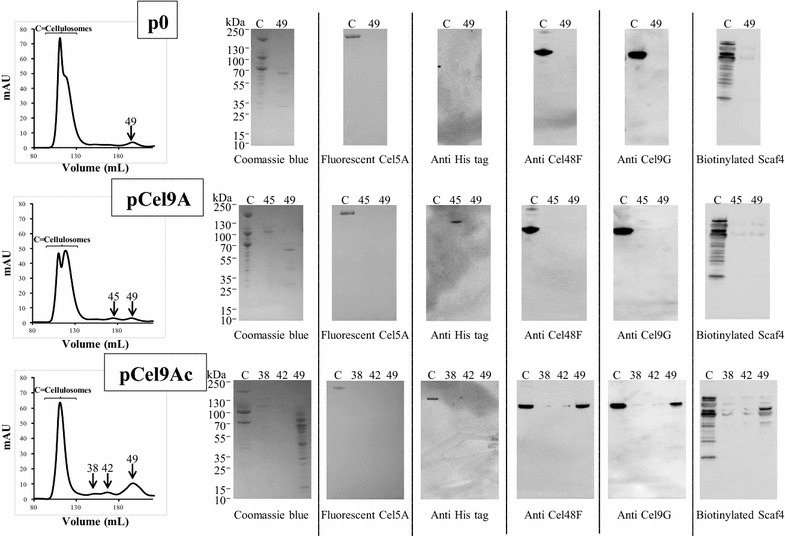
Analyses of cellulosomes and cellulose-bound proteins synthesized by the various *R. cellulolyticum* recombinant strains. Cellulose-bound proteins and cellulosomes were isolated by filtration of 6-day cultures of the various recombinant strains in basal rich medium containing 5 g/L of microcrystalline cellulose (Sigmacell 20). The left panel shows the size exclusion profile of the cellulose-bound fraction produced by the control strain (*top*), the strain carrying pCel9A (*middle*) and the strain harboring pCel9Ac (*bottom*). The ordinate “mAU” designates milli Absorbance Unit at 280 nm. The fractions analyzed by SDS-PAGE and western blots in each chromatogram are indicated. The other panel shows from *left* to *right* SDS-PAGE (stained using *Coomassie blue*), and western blot analyses using fluorescent Cel5A (for detection of the scaffoldin CipC), anti-His tag, anti-Cel48F, anti-Cel9G, and biotinylated hybrid scaffoldin Scaf4 (for detection of dockerin-containing proteins), respectively. The staining procedure or probes used for western blot analyses are indicated at the bottom of each gel. *Lane* C designates the cellulosomes; lanes 38 (observed for strain carrying pCel9Ac), 42 (observed for strain carrying pCel9Ac), 45 (observed for strain carrying pCel9A) and 49 (observed for all strains) designate the corresponding peaks generated during the size exclusion chromatography (*left panel*). For each series of three pictures, the *top photo* corresponds to the control strain, the *middle photo* designates the strain carrying pCel9A, and the *bottom picture* designates the strain harboring pCel9Ac.

The gel filtration profile obtained for the control strain (Fig. 6, top) exhibited two large overlapping peaks of high molecular mass and a third small peak of low molecular mass proteins called fraction 49. As shown in the SDS PAGE analysis of the various fractions (Fig. 6, top), the large overlapping peaks displayed the typical electrophoretic profile of the *R. cellulolyticum* cellulosomes [11], whereas fraction 49 contained a few free proteins whose molecular masses range from 30 to 65 kDa.

The strain carrying pCel9A generated a similar chromatographic profile of cellulose-bound proteins (Fig. 6, middle) with cellulosomes spread into two large overlapping peaks, and a peak “fraction 49”, but an additional small peak called fraction 45 was observed. The analysis of this extra peak by SDS-PAGE (Fig. 6, middle) indicated that it contains a single major protein exhibiting a molecular mass of around 105 kDa. The large overlapping peaks corresponding to the cellulosomes and the peak fraction 49 displayed protein compositions identical to that of the control strain.

In contrast, the gel filtration profile observed for the strain carrying the vector pCel9Ac deviated significantly compared to the two other recombinant strains. A single large peak slightly shifted towards higher molecular mass was obtained for the cellulosomal fraction, whereas the peak corresponding to free proteins (Fraction 49) was much larger than in the case of the two other strain (Fig. 6, bottom). Two supplementary small peaks, called fractions 38 and 42 were also observed. The SDS-PAGE analysis (Fig. 6, bottom) indicated that the cellulosomes contain an extra protein of molecular mass around 110 kDa compared to the cellulosomes produced by the other strains. The most striking difference, however, concerned the peak Fraction 49 which contains many more proteins than in the case of the strains carrying either p0 or pCel9A. The additional peaks 38 and 42 were found to contain small amounts of large proteins.

Western blot analysis using the fluorescently labeled *R. cellulolyticum* cellulosomal cellulase Cel5A [15] as a probe confirmed that the major protein of molecular mass around 170 kDa observed on top of each cellulosomal lane is indeed the scaffoldin CipC (Fig. 6). Using an anti-His tag, the wild-type Cel9A was detected in peak fraction 45 for the strain carrying pCel9A (Fig. 6, middle), whereas the engineered form bearing a dockerin was exclusively detected in the cellulosomal lane of the strain containing pCel9Ac (Fig. 6, bottom), thereby showing that Cel9Ac was fully integrated in the cellulosomes. Interestingly, western blot analyses using antisera raised against Cel48F or Cel9G detected the corresponding enzymes in the peak fraction 49 of the strain carrying pCel9Ac, whereas these cellulases were exclusively found in the cellulosomal lanes of the two other recombinant strains. Furthermore, a number of proteins found in the peak fraction 49 of the strain carrying pCel9Ac contain dockerins as they were probed by the biotinylated hybrid scaffoldin Scaf4 (Fig. 1) harboring the *R. cellulolyticum* cohesin 1 (Fig. 6, bottom). In contrast none of the proteins found in fraction 49 (Fig. 6, top and middle) of the two other recombinant strains reacted with the hybrid scaffoldin. Finally, the large proteins found in peaks 38 and 42 in the case of the strain carrying pCel9Ac failed to unequivocally react with the various probes, and remain to date, unidentified. Altogether, these data suggest that the incorporation of Cel9Ac in the cellulosomes induced an important release of regular cellulosomal components, including the major cellulase Cel48F.

**Fig. 7:**
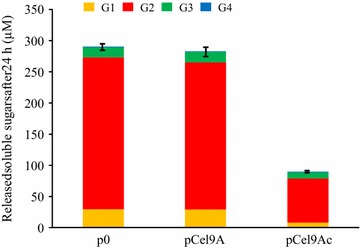
Avicelase activity of the purified cellulosomes produced by the various recombinant *R. cellulolyticum* strains. The purified cellulosomes were assayed at 8 mg/L on 3.5 g/L microcrystalline cellulose for 24 h at 37°C. Released soluble sugars were identified and quantified by HPAEC-PAD. The recombinant strains carrying either p0, pCel9A or pCel9Ac are indicated at the bottom of each bar. G1, G2, G3 and G4 designate glucose, cellobiose, cellotriose and cellotetraose, respectively. The data show the mean of three independent experiments and the *bars* indicate the standard deviation.

The cellulosomes purified by size exclusion chromatography, were also concentrated and assayed at the same concentration on microcrystalline cellulose at 3.5 g/L (Fig. 7). It appeared that the cellulosomes produced by either the control strain or the strain carrying the vector pCel9A displayed the same activity, whereas the cellulosomes synthesized by the strain that produces Cel9Ac exhibited a 3.2-fold lower Avicelase activity. The proportion of released cellodextrins by the three cellulosomes was, however, similar with cellobiose being the most abundant (78–84%), followed by glucose (9–10%) and cellotriose (5–11%).

Thus, the incorporation of Cel9Ac and/or the release of regular cellulosomal components induced a drastic decrease of the activity of the cellulolytic complexes on microcrystalline cellulose.

### Metabolic productivity of *R. cellulolyticum* [pCel9A] and *R. cellulolyticum* [p0] on cellulose.

**Fig. 8:**
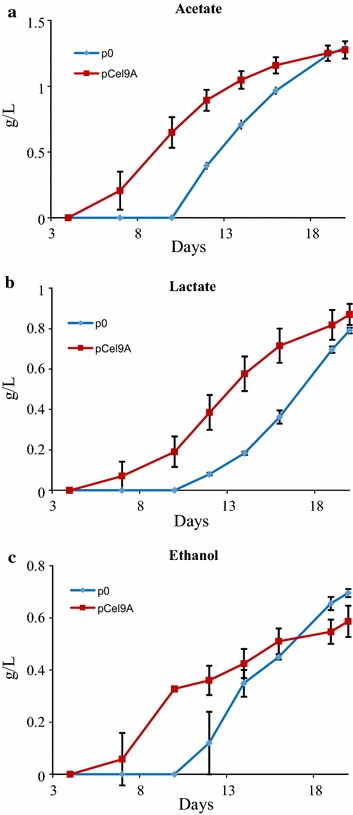
Production of acetate (**a**), lactate (**b**) and ethanol (**c**) by the recombinant strains of *R. cellulolyticum* carrying pCel9A and p0 grown on microcrystalline cellulose. Forty-five-mL mineral media supplemented with Sigmacell 20 microcrystalline cellulose (5 g/L) was inoculated (1/90) with cellobiose-grown cultures of the various recombinant strains. 0.5-mL samples were taken at specific times, centrifuged, and 20-µL aliquots of the supernatant were analyzed by HPLC for ethanol, lactate and acetate content. The data show the mean of three independent experiments and the *bars* designate the standard deviation. The pellet was also analyzed for residual cellulose content by HPAEC-PAD after complete hydrolysis into glucose using sulfuric acid, as well as total protein content using the Lowry method, and the data are reported in Additional file 1.

The production of ethanol, lactate and acetate by the strain secreting the free Cel9A and the control strain when grown on microcrystalline cellulose was monitored, and the data are reported in Fig. 8. As expected, the three major metabolic products were released earlier by *R. cellulolyticum* [pCel9A], compared to the control strain. This observation is indeed consistent with the fact that the Cel9A-secreting strain exhibited a shorter lag phase and consequently an exponential phase of growth occurring sooner on the crystalline substrate (see Additional file 1). Nevertheless, at the end of the cultivation time (Day 20) similar amounts of ethanol, lactate and acetate were detected for both strains.

## Discussion

The present study shows that despite the vast repertoire of GH9 cellulases available in *R. cellulolyticum* which are systematically detected in the cellulosomes, introduction of an additional heterologous GH9 enzyme can considerably improve the cellulolytic capacity of the anaerobic bacterium.

Indeed, Cel9A was chosen because *L. phytofermentans* is also a mesophilic organism and for the reason that the enzyme displays an original organization, which is not found among the various GH9 synthesized by *R. cellulolyticum* or any cellulosome-producing bacteria known to date. In particular, the *L. phytofermentans* cellulase harbors two X2 modules laying between the CBM3 domains. These modules which exhibit an immunoglobulin-like fold with two β-sheets packed against each other [41], are quite common in scaffoldins produced by mesophilic bacteria such as *R. cellulolyticum* [14], *Clostridium cellulovorans* [42], *Clostridium josui* [43] or *Ruminiclostridium papyrosolvens* (formerly known as *Clostridium papyrosolvens* [6]), but to our knowledge they are not found in plant cell wall degrading enzymes except in Cel9A. Their function(s) in bacterial scaffoldins remain(s) unclear as these modules are not involved in the major functions of the scaffoldins: the binding to cellulose (accomplished by the CBM3a) and the binding of the cellulosomal catalytic subunits (performed by the cohesins). It was, however, hypothesized in the case of *C. cellulovorans* that these modules may be involved in the attachment of the cellulosomes to S-layer homologous (SLH) proteins at the surface of the cells [44]. Besides, they were found to facilitate the heterologous secretion of *R. cellulolyticum* cellulases in *Clostridium acetobutylicum* [45]. Their role in Cel9A still needs to be elucidated but they may be involved in the noticeably high activity of this cellulase on crystalline cellulose. As mentioned above, Cel9A is significantly more efficient on microcrystalline cellulose than any of the known cellulases produced by *R. cellulolyticum*. This high activity is consistent with the pivotal role played by this enzyme in the free cellulolytic system of *L. phytofermentans*, which is apparently composed of only few different cellulases [34].

The introduction of Cel9A as a free or as a cellulosomal component had different impacts on the cellulolytic capacities of *R. cellulolyticum*. The integration of the heterologous cellulase into the cellulosomes severely hindered the activity of the complexes, and consequently led to a recombinant strain, which is no longer able to completely metabolize the cellulose initially present in the culture medium. The reduced activity of the cellulosomes is probably mainly due to the substantial release of regular cellulosomal components including the major cellulase Cel48F observed in vivo. As free enzymes, the cellulosomal cellulases of *R. cellulolyticum* are known to be much less efficient on crystalline cellulose, since they no longer benefit from the “proximity” and “substrate targeting” effects generated by their incorporation in cellulosomes [38, 39, 46]. In addition, the presence of several Cel9Ac per scaffoldin may also hinder its activity by inducing some competition. Altogether, these data also indicate that disturbing the naturally occurring equilibrium between cellulosomal enzymes within the complexes can be detrimental, even if a highly active exogenous cellulase is integrated. Nevertheless, on both filter paper and microcrystalline cellulose, this recombinant strain proved to be more efficient compared to the control strain during the first 8–12 days of cultivation. The causes of this phenomenon remain unclear but one may speculate that the *cel9Ac* gene is constitutively expressed thanks to the P_*thl*_ promoter, whereas the expression of the large *cip*-*cel* operon which encodes the scaffoldin and the major cellulosomal cellulases is induced by cellulose and repressed by cellobiose [7, 47]. Thus at the beginning of the culture on minimum medium, an excess of Cel9Ac may be synthesized and secreted as a free enzyme degrading efficiently the cellulosic substrate. At a later stage of the cultivation the scaffoldin CipC is produced in sufficient amount and all heterologous enzymes are trapped in the cellulosomes while a large fraction of regular cellulosomal components can no longer integrate the complexes, thereby causing almost an interruption of the cellulose depolymerisation. This hypothesis is not in contradiction with the fact that no Cel9Ac was detected in the free state after 6 days of culture in rich basal medium supplemented with cellulose (Fig. 6, bottom panel). In this particular rich medium the culture reaches the stationary phase much earlier compared to minimum medium, and the quantity of cellulosomes produced is usually maximal around day 6 as shown in Fig. 6 (left panel) [11].

**Fig. 9:**
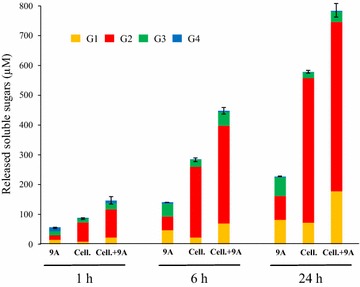
In vitro combination of Cel9A and purified cellulosomes on Avicel 3.5 g/L. 9A designates purified Cel9A (from *E. coli*) at 10.5 mg/L. Cell. corresponds to purified cellulosomes from *R. cellulolyticum* carrying p0 by gel filtration at 10.5 mg/L. Cell. + 9A designates a mixture of 10.5 mg/L of Cel9A and 10.5 mg/L of purified cellulosomes. The soluble sugars released after 1, 6 and 24 h of incubation at 37°C (incubation time indicated at the bottom) were identified and quantified by HPAEC-PAD. G1, G2, G3 and G4 designate glucose, cellobiose, cellotriose and cellotetraose, respectively. The data show the mean of two independent experiments and the *bars* indicate the standard deviation.

In contrast, the cellulolytic capacity of the recombinant strain secreting free Cel9A was improved on both filter paper and microcrystalline cellulose. An important consumption of cellulose occurred sooner compared to the control strain and lead to an early exponential phase of growth. This phenomenon is probably due to both the elevated cellulase activity of Cel9A and its continuous production and secretion thanks to the P_*thl*_ promoter controlling the expression of its gene. Thus, fermentable cellodextrins in sufficient amounts to support a bacterial growth became available sooner for this recombinant strain. Furthermore, one cannot rule out the possibility that some of the soluble sugars rapidly released by Cel9A from cellulose may also trigger an early induction of the expression of the *cip*-*cel* operon encoding the major cellulosomal components, since this operon as mentioned above, is known to be induced by the presence of cellulose, but the exact nature of the inducer, possibly a cellodextrin, remains to be determined [7]. The secretion of free Cel9A had no apparent impact on the protein composition and activity of the cellulosomes. Thus the concurrent production of a fully functional aggregated system and the crucial cellulase of a free enzyme system triggered an enhanced degradation of the cellulose. This improved cellulolytic capacity suggests that Cel9A did not compete with the resident cellulosomes during cellulose hydrolysis. This hypothesis was assessed by measuring the Avicelase activity of a combination of purified cellulosomes and free Cel9A at the same concentration. As seen in Fig. 9, the mix exhibited the same activity (±5%) than the calculated sum of individual activities, thereby demonstrating that in vitro both enzyme systems neither compete nor act synergistically during cellulose hydrolysis. Most probably, the CBM3b hosted by Cel9A and the CBM3a harbored by the scaffoldin CipC which mediates the binding of the cellulosome to the cellulose do not target the same sites on the substrate.

Finally, the improved capacity of the *R. cellulolyticum* strain secreting Cel9A to depolymerize the crystalline cellulose was accompanied by an early production of ethanol, lactate and acetate compared to the control strain.

## Conclusions

The present study identified Cel9A from *L. phytofermentans* as an attractive enzyme to improve the cellulolytic capacities of a cellulosome-producing bacterium, and concomitantly trigger a precocious production of valuable chemicals, when it is secreted as a free enzyme. Our results also showed that this enzyme originating from a free enzyme system neither competes nor acts synergistically with the cellulosomes during cellulose hydrolysis when the heterologous cellulase is secreted in the free state, whereas its integration in the cellulosomes in vivo has a rather negative impact on the activity of the cellulolytic complexes and the ability of the recombinant strain to degrade cellulosic substrates.

## Methods

### Strains and plasmids

*R. cellulolyticum* H10 ATCC 35319 and *L. phytofermentans* DSM 18823 were used in the present study. The BL21(DE3) *Escherichia coli* strain (Novagen, Madison, WI) was used as production strain for the various forms of Cel9A. The plasmid pET28-Cel9A was obtained by amplification of the DNA encoding the mature form of Cel9A (a list of primers used is provided in Additional file 2) from the genomic DNA of *L. phytofermentans* DSM 18823, and subsequent cloning at the NcoI/XhoI sites of pET28a (Novagen). The vectors pET28-Cel9Ac and pET28-Cel9At which encode Cel9A bearing at the C-terminus the dockerin of Cel48F from *R. cellulolyticum* and the dockerin of CelS from *R. thermocellum*, respectively, were obtained by overlap extension PCR, and subsequent cloning of the final amplicon was performed as described above in pET28a. Positive clones were verified by DNA sequencing.

The vector pCel9A was constructed by amplification of the gene encoding Cel9A and subsequent cloning of the amplicon at the BamHI/NarI sites of the vector pSOS952, a pSOS95 [48] derivative containing two *lac* operators upstream and downstream of the *thl* promoter [49]. Construction of the vector pCel9Ac, which encodes Cel9A appended with the C-terminus dockerin of Cel48F from *R. cellulolyticum* required an overlap extension PCR as described above and subsequent cloning of the engineered DNA at the BamHI/NarI sites of the vector pSOS952. The empty vector p0, which was kindly provided by S. Perret, is derived of pSOS952 with the entire expression cassette (P_*thl*_-*adc*-*ctfA*-*ctfB*) deleted.

### Transformation of *R*. *cellulolyticum*

Wild-type strain of *R. cellulolyticum* (H10) was electrotransformed as previously described [50], with pCel9A, pCel9Ac and p0 treated with MspI methylase. Erythromycin resistant clones were isolated under the anaerobic atmosphere of a glove box (N_2_-H_2_, 95:5 [vol/vol]), on solid basal medium supplemented with 2 g/L of cellobiose, 15 g/L of agar, and 10 mg/L of erythromycin. Plates were incubated in anaerobic jars under 2.10^5^ Pa of an N_2_-CO_2_ (80:20 [vol/vol]) atmosphere.

### Media and growth conditions

*E. coli* BL21(DE3) strains carrying pET28a derivatives were grown at 37°C in lysogeny broth supplemented with 50 mg/L of kanamycin. *R. cellulolyticum* H10 was grown anaerobically at 32°C in basal rich medium containing erythromycin at 10 mg/L and supplemented with cellobiose (2 g/L; Sigma-Aldrich, St Louis, MO, USA) for strain constructions or microcrystalline cellulose (5 g/L; Sigmacell cellulose type 20 from Sigma-Aldrich) for cellulosome purifications. Minimal medium [7] containing erythromycin at 10 mg/L and microcrystalline cellulose (5 g/L) was used for bacterial growth study, determination of cellulose consumption and estimation of soluble sugars content. Alternatively, *R. cellulolyticum* was also grown on minimal medium containing erythromycin (10 mg/L) and a strip of filter paper no 1 (7 g/L, Schleicher and Schuell, Dassel, Germany) in Hungate tubes for visualization of the paper stripe degradation.

### Determination of bacterial growth and cellulose consumption on microcrystalline cellulose-containing medium

During cultivation on microcrystalline cellulose, 0.5-mL samples were taken at specific times and centrifuged at 4°C (10,000 *g*) for 10 min. Ten microlitres of supernatant was mixed with 190 µL of distilled water and 50 µL of 0.5 M NaOH, and subsequently analyzed for soluble sugars content by high-pressure anion exchange chromatography coupled with pulsed amperometric detection (HPAEC-PAD, see below). The pellet was resuspended in 200 µL of 1% SDS (w/v) prior boiling for 15 min. The sample was again centrifuged at 4°C (10,000 *g*) for 10 min, and the supernatant served to determine the total protein content using the Lowry method [40]. The pellet was mixed with 500 µL of 12 M H_2_SO_4_ and incubated for 1 h at 37°C under mild shaking. Twenty microlitre of each sample were then mixed with 220 µL of distilled water, and the diluted samples were autoclaved for 1 h at 120°C. After cooling down, 50 µL of 10 M NaOH were added, and the sample was centrifuged at 10,000 *g* for ten min at room temperature. Ten microlitres of supernatant was mixed with 190 µL of distilled water and 50 µL of 0.5 M NaOH, prior analysis of glucose content by HPAEC-PAD (see below).

### Cellulosomes and cellulose-bound proteins purifications and analyses

Cellulosomes and other proteins bound to the residual cellulose were purified from 6-day old cultures of recombinant *R. cellulolyticum* strains in 0.8-L basal rich medium containing 10 mg/L erythromycin and microcrystalline cellulose (5 g/L) as previously described [11]. Briefly, the culture was filtered on 2.7 µm glass filter (Whatman GF/D membrane), and subsequently washed with 50–12.5 mM potassium phosphate buffer (pH 7.0) to remove the cells. One hundred mL of distilled water was used to elute the cellulosomes and the proteins specifically bound to the cellulose. The water-eluted fraction was centrifuged at 4°C for 20 min (10,000 *g*) and subsequently concentrated by ultrafiltration on a membrane displaying a 30-kDa cut off (Sigma-Aldrich), washed with fifty mL of distilled water and concentrated to 2 mL, in a 50-mL ultrafiltration Amicon cell (Merck-Millipore, Billerica, MA, USA). The concentrated sample was subjected to size exclusion chromatography on a Superdex 200 10/300 GL resin (GE Healthcare, Uppsala, Sweden) equilibrated in 20 mM Tris–HCl pH 7.8, 150 mM NaCl and 1 mM CaCl_2_. The various obtained fractions corresponding to the cellulosomes and free proteins were concentrated and dialyzed against 10 mL Tris–HCl pH 8, 1 mM CaCl_2_ by ultrafiltration on vivaspin 20 (cut off 10 kDa, Sartorius, Göttingen, Germany) to 500 µL. The protein concentration of the concentrated samples was determined by the Lowry method. The concentrated samples were analyzed by mixing 10-µL of the samples (corresponding to 8 µg of proteins for the cellulosomal fractions) with 5 µL of denaturing buffer prior boiling for 5 min. Boiled samples were subjected to denaturing polyacrylamide gel electrophoresis (SDS-PAGE) using Bio-Rad (Hercules, CA, USA) precast Gels (gradient 4–15%), and by western blot analyses after transfer on nitrocellulose (Hybond, GE Healthcare) using antisera raised against six His tag, Cel9G and Cel48F. Cel5A labeled with Alexa Fluor 488 succinimidyl ester dye (Protein Labeling Kit, Invitrogen, Carlsbad, NM, USA) and biotinylated Scaf4 were also used to probe the scaffoldin CipC and dockerin-containing proteins, respectively.

Determination of the Avicelase activity of the purified cellulosomes was performed as follows: the filtration fractions corresponding to purified cellulosomes were mixed, concentrated and dialyzed against 10 mL Tris–HCl pH 8, 1 mM CaCl_2_ by ultrafiltration on vivaspin 20 (cut off 10 kDa) to 500 µL. The protein concentration of the concentrated samples was determined by the Lowry method, and the molar concentration of the cellulosomes was estimated using an average molecular mass of 600 kDa.

### Protein production in *E. coli* and purification

The production and purification of Cel48F, Scaf4, Scaf2, cohesin 1 from *R. cellulolyticum* and cohesin 2 from *R. thermocellum* were formerly described [39].

The BL21(DE3) overproducing Cel9A, Cel9Ac and Cel9At were grown in 2.5-L flasks (Nalgene-Thermo Fisher Scientific, Waltham, MA, USA) at 37°C in lysogeny broth supplemented with glycerol (12 g/L) and kanamycin (50 mg/L) until *A*_600_ = 1.5. To prevent the formation of inclusion bodies, the cultures were then cooled down and induction of the expression was performed overnight at 18°C with 50 µM isopropyl-thio-β-d-galactoside. After 16 h of induction, the cells were harvested by centrifugation (3,000 *g*, 15 min), resuspended in 30 mM Tris–HCl pH 8.0, 1 mM CaCl_2_, supplemented with few mg of DNAse I (Roche, Mannheim, Germany), and broken in a French press. The crude extract was centrifuged 15 min at 10,000 *g* and loaded on 2 mL of nickel-nitrilotriacetic acid resin (Qiagen, Vanloo, The Netherlands) equilibrated in the same buffer. The proteins of interest were then eluted with 100 mM imidazole in 30 mM Tris–HCl pH 8.0, 1 mM CaCl_2_. The purification of the recombinant proteins was achieved on Q-Sepharose fast flow (GE Healthcare) equilibrated in 30 mM Tris–HCl pH 8.0, 1 mM CaCl_2_. The proteins of interest were eluted by a linear gradient of 0-500 mM NaCl in 30 mM Tris–HCl pH 8.0, 1 mM CaCl_2_. The purified proteins were dialyzed by ultrafiltration against 10 mM Tris–HCl pH 8.0, 1 mM CaCl_2_, and stored at −80°C. The concentration of the proteins was estimated by absorbance at 280 nm using the program ProtParam tool (http://www.expasy.org/tools/protparam.html).

### Verification of complex formation

Scaf2-, Scaf4- and cohesin-based complexes were verified by non-denaturing PAGE. Interacting protein components (enzymes bearing a dockerin and scaffoldin or cohesin) were mixed at a final concentration of 10 µM at room temperature in 20 mM Tris-maleate pH 6.0, 1 mM CaCl_2_, and 4 µL were subjected to native PAGE (4–15% gradient) using a Phastsystem apparatus (GE Healthcare).

### Enzyme, hybrid minicellulosomes and cellulosomes activity

Activity on CarboxyMethyl Cellulose (CMC, medium viscosity, Sigma-Aldrich) was performed by mixing 4 mL of substrate solution at 10 g/L in 50 mM potassium phosphate buffer pH 6.0, 1 mM CaCl_2_, 0.01% (w/v) NaN_3_ with 40 µL of an appropriate enzyme dilution, at 37°C. Aliquots (500 µL) were pipetted at specific intervals, cooled down in ice and analyzed for reducing sugar contents by the Park and Johnson [51] method using glucose as the standard.

Activity on Avicel (PH 101, Fluka, Buchs, Switzerland) at 3.5 g/L was performed similarly (final volume of 4 mL) under mild shaking (70 rpm) at 37°C at a final enzyme or complex concentration of 0.1 µM except that 800-µL aliquots were pipetted at specific intervals and centrifuged for 10 min (10,000 *g*) at 4°C. The supernatants were analyzed for reducing soluble sugar contents using the Park and Johnson method and HPAEC-PAD (see below). Determination of the Avicelase activity of the purified cellulosomes was performed similarly except that the final concentration of the complexes was 8 mg/L. The activity on microcrystalline cellulose of a combination of purified cellulosomes and Cel9A was performed as described above except that the final concentrations of the cellulosomes and Cel9A were both set at 10.5 mg/L.

### HPAEC-PAD analyses

Identification and quantification of the released soluble sugars by HPAEC-PAD were performed in a Dionex ICS 3000 (Sunnyvale, CA, USA) equipped with a pulsed amperometric detector. 200 µL of sample (or appropriate dilution of samples in distilled water) was mixed with 50 µL of 0.5 M NaOH and 25 µL were applied to a Dionex CarboPac PA1 column (4 × 250 mm) preceded by the corresponding guard column (4 × 50 mm) at 30°C. Sugars were eluted with the buffers 0.1 M NaOH and 0.5 M sodium acetate +0.1 M NaOH as the eluents A and B, respectively. For glucose quantification the following multi-step procedure was used: isocratic separation (5 min, 95% A + 5% B), column wash (2 min, 99% B) and subsequent column equilibration (2.5 min, 95% A + 5% B). For analysis of cellodextrins, the same A and B buffers were used but the multi-step procedure was as follows: isocratic separation (5 min, 95% A + 5% B), separation gradient (8 min, 10–37% B), column wash (2 min, 99% B) and subsequent column equilibration (2.5 min, 95% A + 5% B). The flow rate was kept at 1 mL/min in all cases. Injection of samples containing glucose, cellobiose, cellotriose, cellotetraose, cellopentaose (Sigma-Aldrich) and cellohexaose (Seikagaku, Tokyo, Japan) at known concentrations (ranging from 5 to 100 µM) was used to identify and quantify the released sugars.

### Analyses of metabolic products

Five hundred microlitre samples were taken at specific intervals from cultures of *R. cellulolyticum* carrying pCel9A and p0 on mineral medium supplemented with erythromycin (10 mg/mL) and microcrystalline cellulose (5 g/L). The samples were centrifuged for 10 min at 10,000 *g*, and the pellets were analyzed for total protein and residual cellulose contents as described above (the data are reported in Additional file 1). The supernatants were centrifuged again using the same conditions and the final supernatants were filtered through 0.22 µM microfilters (Sartorius). Acetate, ethanol and lactate were measured in duplicate using high performance liquid chromatography (HPLC) analysis (Agilent 1200 series, Massy, France) essentially as previously described [52]. The separations were performed on a Bio-Rad Aminex HPX-87H column (300 × 7.8 mm), and detection was achieved using either a refractive index measurement or a UV absorbance measurement (210 nm). The operating conditions were as follows: temperature, 48°C; mobile phase, H_2_SO_4_ (5 mM); and flow rate 0.5 mL/min.

## Additional files

Additional file 1: Cellulose consumption and growth of the recombinant *R. cellulolyticum* strains carrying pCel9A and p0 on microcrystalline cellulose, that served to monitor the productions of acetate, lactate and ethanol reported in Fig. 8. Additional file 2: Primers used.

## Abbreviations

CBM: carbohydrate binding module
GH: glycoside hydrolase
CMC: carboxymethyl cellulose
Cel9Ac: Cel9A bearing a C-terminal *R. cellulolyticum* dockerin
Cel9At: Cel9A bearing a C-terminal *R. thermocellum* dockerin
HPAEC-PAD: high-pressure anion exchange chromatography coupled with pulsed amperometric detection
HPLC: high-pressure liquid chromatography
PAGE: polyacrylamide gel electrophoresis
G1, G2, G3, G4, and G5: glucose, cellobiose, cellotriose, cellotetraose and cellopentaose, respectively
