# Supplementary material for: Combining free and aggregated cellulolytic systems in the cellulosome-producing bacterium Ruminiclostridium cellulolyticum
Source: Biotechnol Biofuels. 2015 Aug 13;8:114. doi: 10.1186/s13068-015-0301-4 (PMC4533799; doi:10.1186/s13068-015-0301-4)
Supplement: Additional file 1: — Cellulose consumption and growth of the recombinant R. cellulolyticum strains carrying pCel9A and p0 on microcrystalline cellulose, that served to monitor the productions of acetate, lactate and ethanol reported in Fig. 8. [file 13068_2015_301_MOESM1_ESM.pdf]

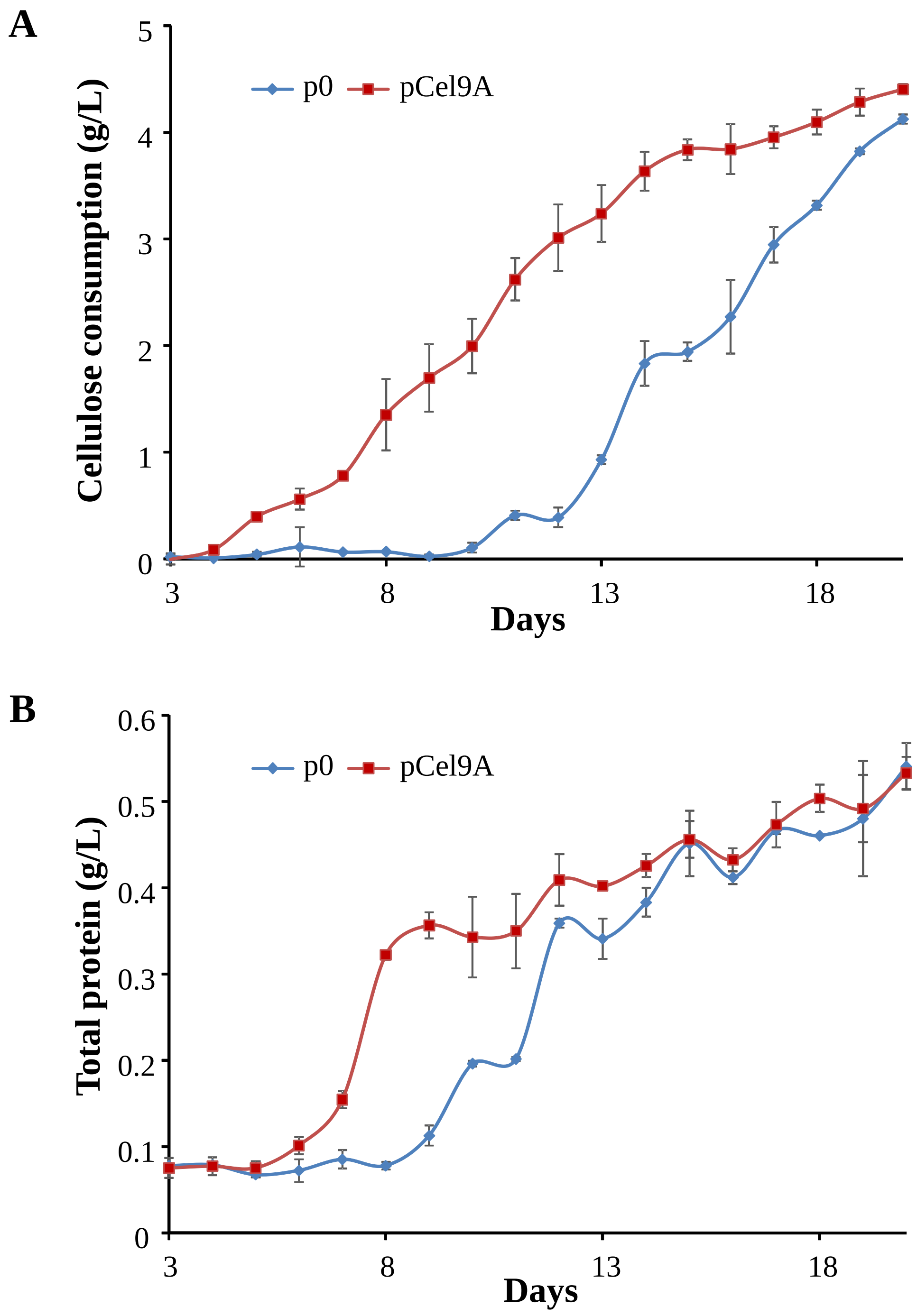

Additional Figure : Cellulose consumption (A) and growth (B) of the recombinant *R. cellulolyticum* strains carrying pCel9A and p0 on microcrystalline cellulose.

The data correspond to figure 8.

Samples were taken at specific times, centrifuged and while the supernatants were analysed for acetate, lactate and ethanol (figure 8), the corresponding pellets were analyzed for residual cellulose content by HPAEC-PAD after complete hydrolysis into glucose using sulfuric acid, and total protein content using the Lowry method. Blue line and diamonds designate the control strain carrying p0; red line and squares correspond to the recombinant strain carrying pCel9A. The data show the mean of three independent experiments and bars indicate the standard deviation.

Additional file 2
