# Supplementary material for: Combining free and aggregated cellulolytic systems in the cellulosome-producing bacterium Ruminiclostridium cellulolyticum
Source: Biotechnol Biofuels. 2015 Aug 13;8:114. doi: 10.1186/s13068-015-0301-4 (PMC4533799; doi:10.1186/s13068-015-0301-4)
Supplement: Additional file 2: — Primers used. [file 13068_2015_301_MOESM2_ESM.pdf]

**Primers used for overproduction in *E. coli* of the various forms of Cel9A**		
Enzyme	Primer Name	Primer Sequence
Cel9A	9Af	5’-GGGGGGCCATGGCGGAAACCAATTATAATTACGGAGAAGC-3’
Cel9A	9Ar	5’-AAAAACTCGAGTGGTTCAACTCCCCAAACTAAAACATCAGA-3’
Cel9Ac	9A-Dcf	5’-CTCTGATGTTTTGGTATGGGGAGTCGAACCAGGTAACGAACCCGCAGGTGGATCA-3’
Cel9Ac	9A-Dcr	5’-TGATCCACCTGCGGGTTCGTTACCTGGTTCGACTCCCCATACCAAAACATCAGAG-3’
Cel9Ac	petrev	5’-CAGCTTCCTTTCGGGCTTTGTTAGCAGC-3’
Cel9At	9A-Dtf	5’-CTCTGATGTTTTGGTATGGGGAGCCGAACCAGGTACTCCTTCTACTAAATTATACGGC-3’
Cel9At	9A-Dtr	5’-GCCGTATAATTTAGTAGAAGGAGTACCTGGTTCGGCTCCCCATACCAAAACATCAGAG-3’
Cel9At	DokeTr	5’-TTATTCTCGAGGTTCTTGTACGGCAATGTAT-3’
**Primers used for production in *R. cellulolyticum* of the various forms of Cel9A**		
Cel9A	9A-952f	5’-GGGGATCCAGAATTTAAAAGGAGGGATTAAAATGAAAAAAATAATAAGTCTTTTATTAGTGATAACAC-3’
Cel9A	9A-952r	5’-CCCCCGGCGCCTTAGTGGTGGTGGTGATGATGTGGTTCGACACCCCAAACTAAAACATC -3’
Cel9Ac	9Ac-952f	See above
Cel9Ac	9A-Dcf	See above
Cel9Ac	9A-Dcr	See above
Cel9Ac	HisNarrev	5’-ATTAAGTGGCGCCCTAGTGGTGGTGGTGGTGGTGCTCGAG -3’

Additional file 1
